# Aerobic Oxidation of 5-Hydroxymethylfurfural (HMF) in Aqueous Medium over Fe-Doped-Poly(heptazine imide) Photocatalysts: Unveiling the Bad Role of Hydroxyl Radical Generation on the Catalytic Performance

**DOI:** 10.3390/molecules28248077

**Published:** 2023-12-14

**Authors:** José B. G. Filho, Ingrid F. Silva, Mamdouh Alafandi, Jabor Rabeah

**Affiliations:** 1 Leibniz Institute for Catalysis (LIKAT Rostock), D-18059 Rostock, Germany; balenagabriel@gmail.com (J.B.G.F.); mamdouh.alafandi@gmail.com (M.A.); 2 Department of Chemistry, Federal University of Minas Gerais (UFMG), Belo Horizonte 31270-901, MG, Brazil; 3 Department of Colloid Chemistry, Max Planck Institute of Colloids and Interfaces, Potsdam Science Park, Am Mühlenberg 1, D-14476 Potsdam, Germany; ingrid.silva@mpikg.mpg.de

**Keywords:** lignocellulose biomass, 5-hydroxymethylfurfural, photocatalysis, carbon nitride, EPR

## Abstract

5-hydroxymethylfurfural (HMF) oxidation in aqueous media using visible photocatalysis is a green and sustainable route for the valorization of lignocellulosic biomass derivatives. Several semiconductors have already been applied for this purpose; however, the use of Poly(heptazine imides), which has high crystallinity and a special cation exchange property that allows the replacement of the cation held between the layers of C_3_N_4_ structure by transition metal ions (TM), remains scarce. In this study, PHI(Na) was synthesized using a melamine/NaCl method and used as precursor to prepare metal (Fe, Co, Ni, or Cu)-doped PHI catalysts. The catalysts were tested for selective oxidation of HMF to 2,5-diformylfuran (DFF) in water and O_2_ atmosphere under blue LED radiation. The catalytic results revealed that the 0.1 wt% PHI(Fe) catalyst is the most efficient photocatalyst while higher Fe loading (1 and 2 wt%) favors the formation of Fe^3+^ clusters, which are responsible for the drop in HMF oxidation. Moreover, the 0.1 wt% PHI(Fe) photocatalyst has strong oxidative power due to its efficiency in H_2_O_2_ production, thus boosting the generation of nonselective hydroxyl radicals (^●^OH) via different pathways that can destroy HMF. We found that using 50 mM, the highest DFF production rate (393 μmol·h^−1^·g^−1^) was obtained in an aqueous medium under visible light radiation.

## 1. Introduction

The use of fossil sources to fill the human necessity for energy and building block molecules has been bringing severe consequences to modern society, such as economic crises, uncontrolled pollution, and worsening of the greenhouse effect [1]. Facing these problems, the authorities have shifted their mindset towards the search for more sustainable alternative carbon sources such as lignocellulosic biomass, the most well-regarded alternative [2]. It can be obtainable in large quantities from agro-industrial wastes, i.e., from non-edible feedstock. Since lignocellulosic biomass is basically composed of cellulose, hemicellulose, and lignin, it is possible to process these polymeric structures in order to obtain extremely valuable platform molecules for a green and oil-independent future [3]. 5-hydroxymethylfurfural (HMF) is listed as a quite important biomass compound [4,5]. It is produced by acid-catalyzed dehydration of glucose, the cellulose monomer, which is the most abundant carbohydrate available in the world [6]. It is possible to transform HMF into many valuable downstream derivatives, among them 2,5-diformylfuran (DFF) and 2,5-Furandicarboxylic acid (FDCA), compounds produced by oxidative routes that have great versatility in synthetic applications to generate fine chemicals and sustainable polymers [5,7].

The catalytic selective oxidation of HMF has been well reported in the literature [8,9]; however, most of the system relies on very expensive noble metals due to their outstanding ability to activate molecular oxygen. Moreover, conducting the catalytic reaction at high temperatures leads to the formation of by-products such as levulinic acid and favors the polymerization of HMF and derivatives into humines [10], which consequently deactivate the catalyst. In this context, heterogeneous photocatalysis is an interesting and green route for HMF conversion since it is possible to use visible light (the largest fraction of sun radiation) as the driving force to trigger redox mechanisms in semiconductors [11].

Carbon-nitride-based materials (g-C_3_N_4_) have received remarkable attention as visible-light-driven photocatalysts due to their electronic and redox properties, thermal stability, low cost, and non-laborious synthesis. Among the various types of carbon nitride available in the literature, Poly(heptazine imides) (PHI) have shown excellent photocatalytic activity for hydrogen evolution reaction [12]. It is a crystalline polymeric material that can accumulate up to 20 times more electrons than conventional g-C_3_N_4_ [13]. Besides that, its layered structure has charge-compensated cations, (–N=C=N^−^M^+^), remaining from the salt used in the synthesis procedure, which are exchangeable, making it a versatile and tunable material [14,15,16]. This cation exchange property in PHI has also been retained as a strategy for developing single-atom/single-site catalysts (SACs) [14,17,18,19]. The synthesis of SACs is extremely advantageous since it is possible to achieve better performance with a minimal amount of metal (highly dispersed active sites) contributing to the high atom economic efficiency [19,20,21,22].

Carbon-nitride-based materials (g-C_3_N_4_) have received remarkable attention as visible-light-driven photocatalysts due to their electronic and redox properties, thermal stability, low cost, and non-laborious synthesis. Among the various types of carbon nitride available in the literature, Poly(heptazine imides) (PHI) have shown excellent photocatalytic activity for hydrogen evolution reaction [12]. It is a crystalline polymeric material that can accumulate up to 20 times more electrons than conventional g-C_3_N_4_ [13]. Besides that, its layered structure has charge-compensated cations, (–N=C=N^−^M^+^), remaining from the salt used in the synthesis procedure, which are exchangeable, making it a versatile and tunable material [14,15,16]. This cation exchange property in PHI has also been retained as a strategy for developing single-atom/single-site catalysts (SACs) [14,17,18,19]. The synthesis of SACs is extremely advantageous since it is possible to achieve better performance with a minimal amount of metal (highly dispersed active sites) contributing to the high atom economic efficiency [19,20,21,22].

Despite the excellent articles on the photocatalytic application of non-noble transition-metal–PHI-based SACs, including those published by Teixeira et al. on photocatalytic C–H activation in hydrocarbons [15,16] and Cai et. al on cross-coupling C-heteroatom reactions [14], these promising materials remain incipient in the photocatalysis literature. Regarding the valorization of lignocellulosic biomass platform molecules (especially HMF) over g-C_3_N_4_-based material, good results rely on heterojunctions due to the poor activity of regular g-C_3_N_4_ and have mainly been obtained when experiments were conducted in organic solvents [23,24,25,26,27]. This is neither sustainable nor technically advantageous since HMF is industrially produced in aqueous media [28,29]. Hence, it is essential to develop a photocatalyst that operates in the aqueous system. Interestingly, there are no publications aiming at the conversion of this substrate using PHI structures. Therefore, the present study is dedicated to the application of doped TM ions on PHI for the oxidation of HMF in the visible spectral range (blue LED), namely, Na, Fe, Co, Ni, and Cu PHI-based catalysts. Moreover, the effect of metal loading, solvents, pH and reaction time on the photocatalytic activity was investigated in depth over 0.1 wt% PHI(Fe), which showed the best photocatalytic activity. In addition, in situ electron paramagnetic resonance (EPR) has been applied to better understand the photocatalytic process and gain deep insights into the reaction mechanism.

## 2. Results and Discussion

### 2.1. Characterizations

**Figure 1:**
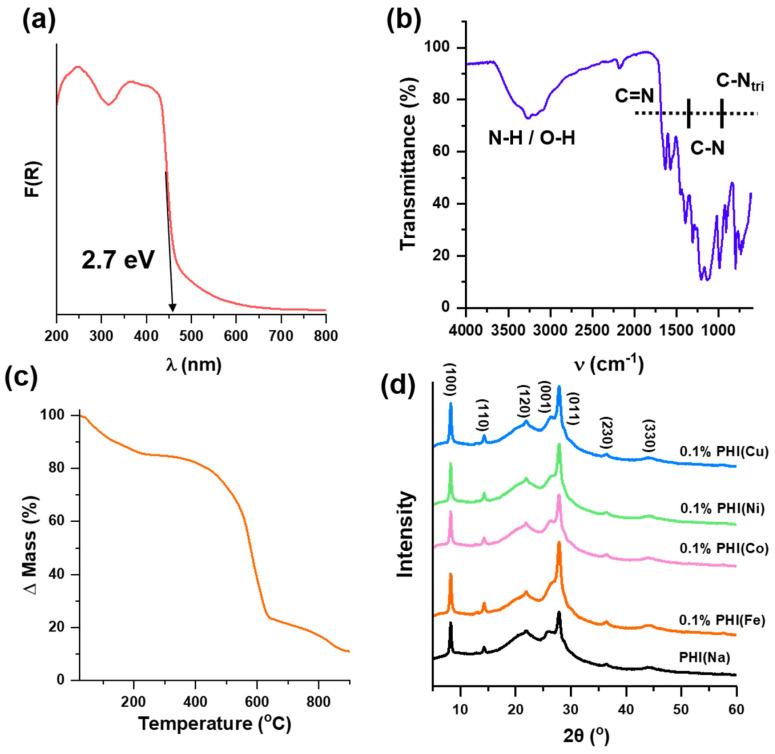
(**a**) Kubelka–Munk function obtained from DRS spectrum of PHI(Na) and band gap calculated by Tauc plot, (**b**) FTIR spectrum, (**c**) thermogram of PHI(Na), and (**d**) XRD powder pattern for PHI(Na), PHI(Fe), PHI(Co), PHI(Ni), and PHI(Cu) 0.1 wt%.

ICP-OES results of the PHI(Na) showed that it contained about 5% residual sodium in its structure due to the molten salt synthetic method. The Kubelka–Munk function obtained from diffuse reflectance spectroscopy (DRS) (Figure 1a) shows that this semiconductor polymer is able to harvest light from the visible spectral range (band gap of 2.7 eV using the Tauc plot method). The metal concentrations used did not significantly change the color of the materials; therefore, only the UV–Vis spectrum of PHI(Na) was included in the discussion. The FTIR spectrum from 500 to 2000 cm^−1^ (Figure 1b) shows several bands; those ranging from 800 to 1100 cm^−1^ are assigned to C–N stretching of the Tri-s-triazine rings of carbon nitride, while those in the 1100–1500 cm^−1^ region are assigned to aromatic C–N stretches. The vibrational transitions over 1500 cm^−1^ are characteristic of aromatic C=N [16,30]. Around 3200 cm^−1^, the N–H stretches of the amino groups overlap with the hydroxyl signal of the adsorbed water on the surface [30]. The thermogravimetric analysis (Figure 1c) shows a mass loss of ~20% until 500 °C, probably associated with adsorbed water and decomposition of the amino groups detected using FTIR spectroscopy. From this temperature point, a drastic drop was observed, suggesting the collapse of the polymeric structure in air. The specific surface area calculated using the BET method is 39 m^2^·g^−1^.

X-ray diffractometry (XRD) (Figure 1d) shows some reflection peaks between 5 and 50° that belong to the (100), (110), (120), (001), (011), (230) and (330) planes of PHI(Na) [16]. These crystalline features do not change when the desired metal is added, even in higher amounts (see Figure S3), indicating that the structure of PHI remains stable after the cation exchange process. The structural ordering can also be observed using the TEM technique (Figure S2a,b), which shows the stacked layers of PHI(Na) and its high crystallinity, as evidenced by the lattice fringes throughout the sample. Most lattice fringes observed in these images refer to the plane (110), ~11 Å [12,18,31]. On the other hand, when SAED was analyzed, the electron diffraction profile also showed the (001) plane with lattice spacing of 3.2 Å, which is attributed to the stacking of the heptazine units in the polymeric structure [12,19,32]. The amount of metal was evaluated using ICP-OES and can be seen in Table S1; the theoretical values are not dissimilar to the real ones, indicating the efficiency of the cation exchange procedure to prepare the PHI materials.

### 2.2. Photocatalytic Oxidation of HMF

**Figure 2:**
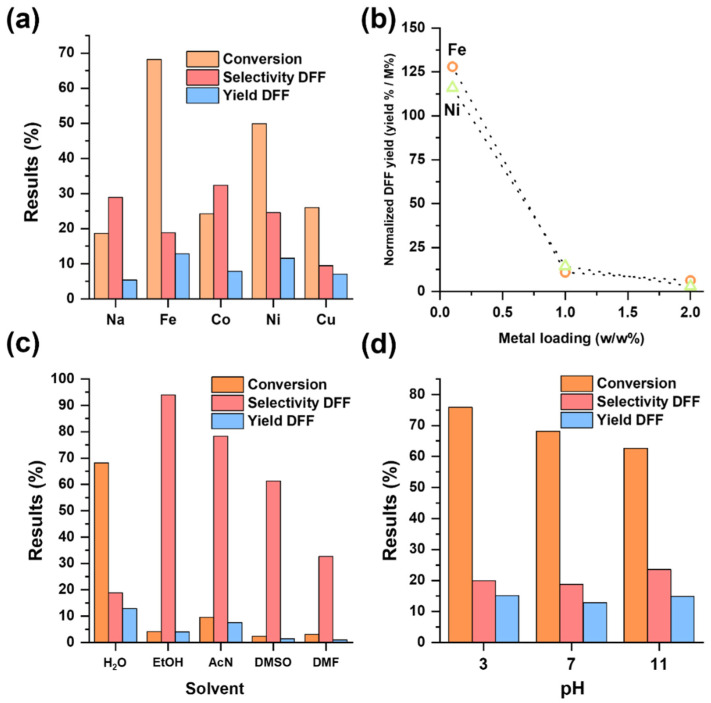
(**a**) Photocatalyst screening results for the pristine PHI(Na) and different 0.1 metals wt% PHI; (**b**) normalized DFF yield using the metal loading for PHI(Ni) and PHI(Fe) in different metal concentrations; (**c**) photolytic results in different solvents and (**d**) different pH values (water as the solvent) using 0.1 wt% PHI(Fe). All the reactions performed in this figure were carried out using 10 mg of photocatalyst, 2 mL of 5 mM HMF solution, a 2 h irradiation time (Blue LED, 456 nm), room temperature (~20 °C) and an O_2_ balloon installed on the top part of the reactor.

The photocatalytic results show that PHI(Na) is the least active catalyst, indicating that the introduction of transition metals into the PHI structure plays a role in the photocatalytic performance. Among PHI-based catalysts, the 0.1 wt% PHI(Fe) showed the best yield for the HMF-to-DFF transformation in 2 h (12.8%) followed by PHI(Ni) (11.5%), PHI(Co) (7.9%), and PHI(Cu) (7.1%). In addition to DFF, 0.1 wt% PHI(Fe) was able to produce FDCA in 4.0% of the yield while 0.1 wt% PHI(Ni) could produce FDCA in only 1.4%. In order to evaluate the effect of the amount of metal in the polymeric matrix of PHI(Fe) and PHI(Ni), 1 and 2 wt% of the metal loadings were also tested in this reaction. It is clear from analyzing the profile of the normalized yield of DFF (yield of DFF divided by the percentage of metal loading added) that the amount of metal influences the yield of the desired product (Figure 2b). There was a sudden drop in the yield values when the amount of metal increased from 0.1 to 1% (over 10 times lower for PHI(Fe)), while this drop was not too significant upon increasing the metal loading from 1 to 2 wt%. It is concluded that the higher metal loadings do not favor DFF production. Due to the high performance of 0.1wt% PHI(Fe), its photocatalytic activities were evaluated for different solvents (Figure 2c). Despite the good selectivity, none of the organic solvents tested achieved as good a yield for the DFF as water. Besides the good selectivity, poor conversions culminated in yields below 10% for DMF, DMSO, AcN and EtOH. This result is promising since the most successful examples published in this reaction are commonly associated with organic solvents [23]. As shown in Figure S4, the PHI is more easily suspended in H_2_O than in AcN,(the best solvents), which was attributed to the ionic nature of the PHI structure [33]. Therefore, the effect of pH on the photocatalytic performance of 0.1wt% of PHI(Fe) catalyst was investigated in an aqueous medium. A slight change in the photocatalytic performance was observed (Figure 2d) when the pH was shifted from neutral to lower and higher values, 3 and 11, respectively. This means that the photocatalyst is robust in this wide range of pH and the proton concentration does not significantly change the reaction pathway. From a technical–sustainable point of view, working at a neutral pH is always better. Therefore, the kinetic data were evaluated at neutral pH over 0.1 wt% PHI(Fe) catalyst.

**Figure 3:**
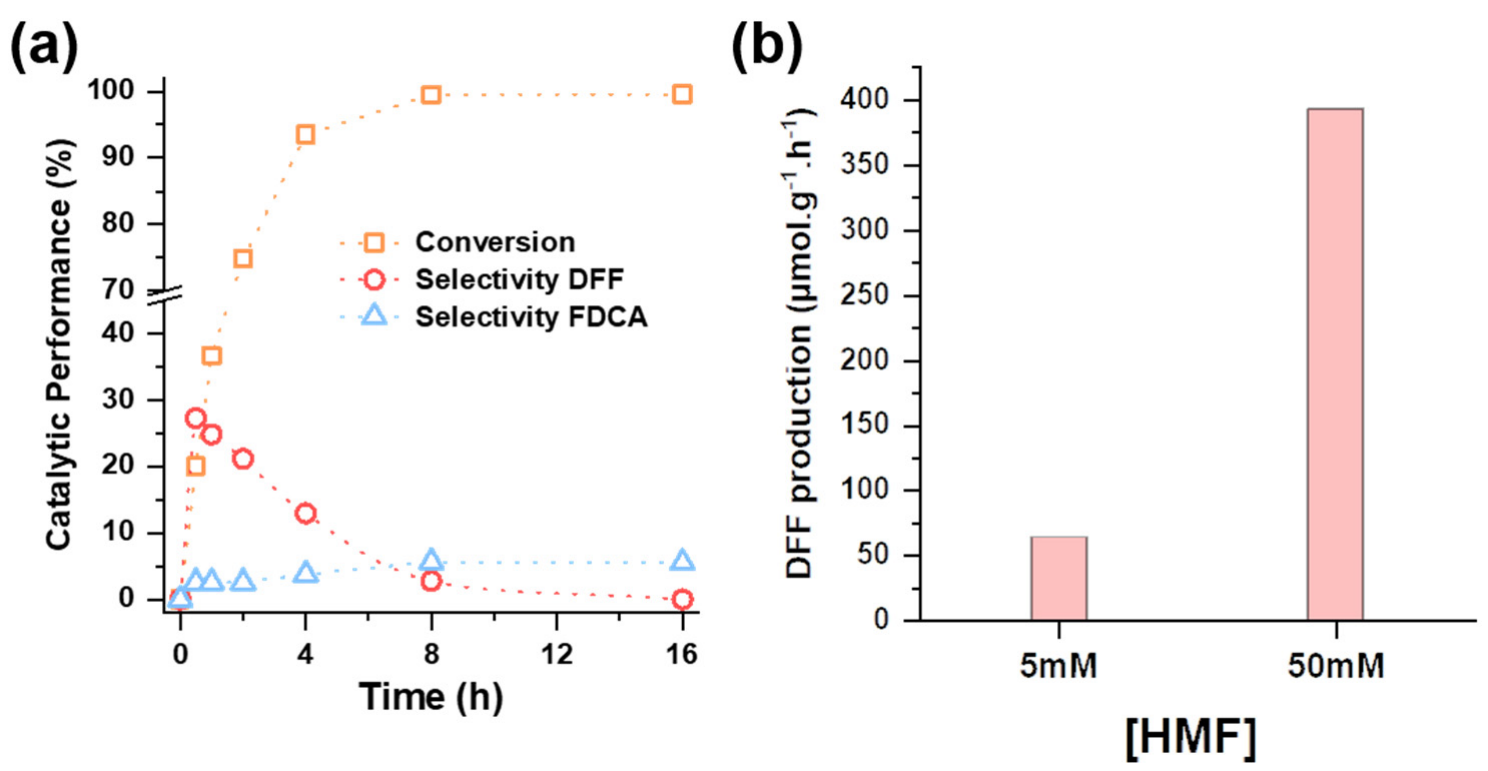
HMF conversion and selectivity to DFF over 0.1 wt% PHI(Fe) (**a**) using 0.5 mM of HMF and (**b**) DFF production rate at different HMF concentrations.

As shown in Figure 3a, the maximum DFF selectivity was obtained during the first 30 min, after which it dropped rapidly while the conversion of HMF evolved continuously. When the conversion practically reaches 100% in 8h, there is almost no DFF in the reaction medium. The FDCA concentration is increased at a very low rate in comparison to the decline in DFF over time, indicating that the rate of DFF formation is much lower than its oxidation to FDCA. Traces of FDCA were observed at all points on the curve, indicating that PHI(Fe) oxidizes DFF to FDCA quickly. A chromatographic run focused on early eluted products revealed that formic acid is detected in two hours of reaction (Figure S5); such a compound is normally produced during severe oxidation of lignocellulosic biomass molecules [34,35]. The over-oxidation caused by this photocatalyst is not surprising since its ability to activate C-H bonds of alkanes is known [15,16]. An attempt to overcome the over-oxidation issue was to substantially increase the HMF concentration, from 5 mM to 50 mM (Figure 3b). The production rate of the DFF was impressively boosted from 64 to 393 μmol·g^−1^·h^−1^, reaching the highest value in the literature when using an aqueous medium and visible light as an energy source. For comparison purposes, literature values are shown in Table S2.

### 2.3. Insights into Surface Structure and Mechanism

The XPS survey spectra for PHI(Na) and 0.1 wt% PHI(Fe) appear to have no difference (Figures S6a and S7a, respectively). The high-resolution spectra for C1s and N1s show some similarities as well; both samples have the regular C1s (C–C: 284.8 eV, C_3_–N: 286.3 eV, N–C=N: 288.2 eV, C(π): ~293 eV) and N1s (C_3_–N: 400.1 eV, N–C=N: 398.6 eV and C–N–H: 401.1 eV) peaks for g-C_3_N_4_ [16,36,37] (Figures S6b,c and S7b,c, respectively). However, the C1s peak at 289.6 eV was detected in the 0.1 wt% PHI(Fe) spectrum (Figure S7b) while being almost undetectable in PHI(Na) (Figure S6b). This peak has been attributed to N=C–O, reinforcing the oxidative character of the Fe sites in the PHI structure. In the case of the N1s spectrum, its deconvolution also showed a sharper peak at higher binding energies (403.4 eV, cyan curve) for 0.1 wt% PHI(Fe) in comparison with PHI(Na) (see Figures S6c and S7c), which is normally assigned to N in an electron-deficient chemical environment, probably close to Fe^3+^ [15]. Despite the noisy spectrum, it was possible to detect photoelectrons emitted in the Fe 2p binding energy region for 0.1 wt% PHI(Fe), but due to the low concentration, it is difficult to extract precise information on this surface species (Figure S7d).

**Figure 4:**
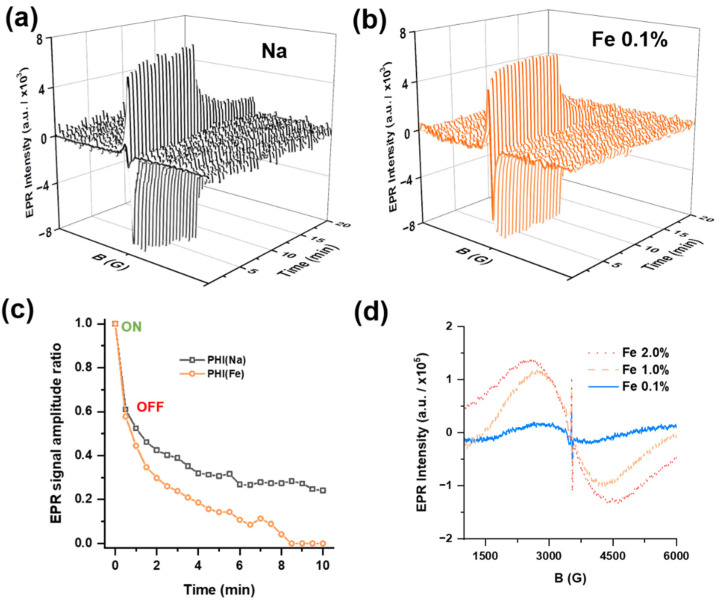
EPR spectra over time recorded during turning the light ON for 10 min and then light OFF for 10 min for (**a**) PHI(Na) and (**b**) 0.1 wt% PHI(Fe); (**c**) EPR signal decay in the dark after turning off the light of the irradiated catalysts (the spectra were normalized to 1 by dividing them to the last signal recorded during light (ON)); (**d**) EPR spectra of 0.1, 1 and 2 wt% PHI(Fe) catalysts measured in a wider magnetic field range.

For a better understanding of the reaction pathway, in-depth ex and in situ EPR investigations were performed. The EPR spectra of PHI(Na) and 0.1, 1 and 2 wt% PHI(Fe) solid catalysts in the dark exhibited a sharp signal at g = 2.004 attributed to the trapping of unpaired electrons in the C_3_N_4_ structure (Figure 4a,b and Figure S8a,b,d) [38]. The EPR signal intensity increased upon in situ illumination of the catalysts due to the increase in electron density in the π conjugated system of C_3_N_4_ [38]. As all signals were normalized by mass and have the same line width peak to peak, a direct comparison can be made using the height of the EPR signal intensities. We found that the increases in the EPR signal intensities during illumination of the catalysts were in the following order: PHI(Na) > 0.1 wt% PHI(Fe) > 1 wt% PHI(Fe) > 2 wt% PHI(Fe). The enhancement of the EPR signal intensity was not strong for 1 and 2 wt% PHI(Fe) catalysts (Figure S8a,b), likely due to the direct transfer of the conduction band photoexcited electrons to the Fe^3+^ centers. Among the photocatalysts, 0.1 wt% PHI(Fe) showed the fastest response to light and almost maintained the signal intensity with time. The time-dependence of the decay of the trapped-photoexcited electrons over PHI(Na) and 0.1 wt% PHI(Fe) was monitored by turning the light off and measuring EPR spectra in the dark over time (for 10 min after irradiation of the catalysts). In the plot of Figure 4c, the EPR signal intensity of PHI(Na) dropped after turning of the light; however, it was still higher than that before the illumination, suggesting that the catalyst can stabilize some of the photoexcited electrons, i.e., it takes a long time for the photoexcited electrons to return to their ground state. Interestingly, the EPR signal intensity of the irradiated 0.1 wt% PHI(Fe) catalyst is similar to that after turning off the light for 10 min, suggesting that some of the photoexcited electrons were trapped by Fe^3+^ sites. This hypothesis was confirmed using time-resolved photoluminescence spectroscopy (Figure S8c), which showed that the photogenerated charges in 0.1 wt% PHI(Fe) have a longer lifetime than in PHI(Na), supporting that Fe^3+^ sites trap the photoexcited electrons and decrease the recombination rate [39]. All PHI(Fe) catalysts exhibited a broad EPR signal due to the formation of Fe^3+^ ferromagnetic clusters (Figure 4d). The intensity of the ferromagnetic signal was more pronounced in the 2 wt% PHI(Fe) catalyst, indicating that increasing the Fe loading leads to the aggregation of Fe ions into ferromagnetic clusters.

**Figure 5:**
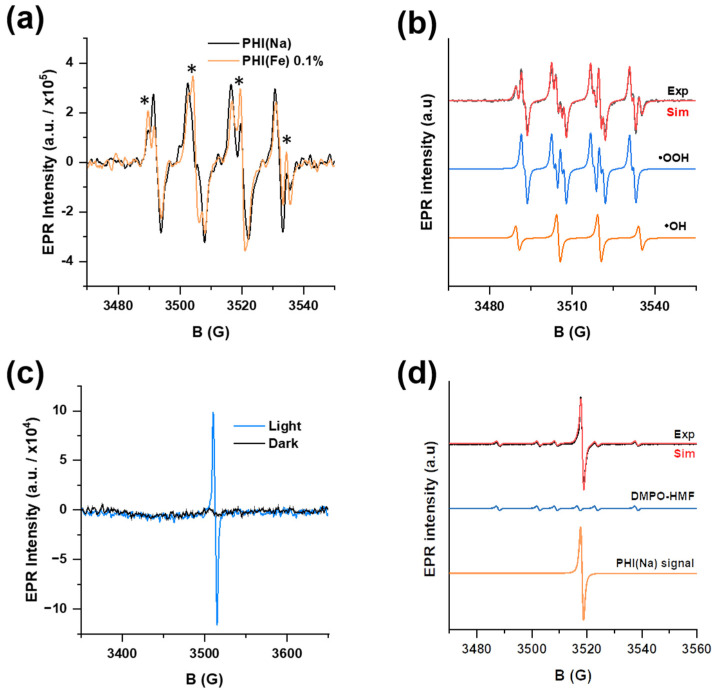
(**a**) EPR spectra of DMPO spin adducts for PHI(Na) and 0.1 wt% PHI(Fe) in aqueous 5 mM HMF solution and O_2_ atmosphere (* EPR signal of the DMPO-OH spin adduct); (**b**) experimental and simulated EPR spectra of these spin adducts; (**c**) EPR spectra of DMPO test in aqueous 5 mM HMF solution in Ar atmosphere (dark and light ON) for 0.1 wt% Fe-PHI; (**d**) experimental and simulated EPR spectra of DMPO spin adduct in 5mM HMF in AcN solution in Ar atmosphere for 0.1 wt% PHI(Fe).

To detect and investigate the nature of the radical species that might be formed during the photocatalytic reactions, DMPO spin trapping experiments were performed. In situ EPR investigations showed that irradiation of PHI(Na) and 0.1 wt% PHI(Na) suspensions in the presence of O_2_-saturated HMF solution leads to the formation of DMPO-OH and DMPO-OOH spin adducts (Figure 5a), confirmed through the EPR simulations of the experimental spectra (Figure 5b) [40,41]. However, carefully looking at the overlapped spectra in Figure 5a (recorded after 2 min of irradiation) revealed that the EPR signal of DMPO-OH over 0.1 wt% PHI(Fe) is more intense than PHI(Na). On the contrary, the signal of the DMPO-OOH spin adduct was slightly more pronounced over PHI(Na). Taking this information into account along with the difference in the EPR signal decay profile in the dark (Figure 4c), it is deduced that the trapped electrons reduced Fe^3+^ to Fe^2+^ and that this redox pair plays a role in the formation of a higher amount of a very powerful oxidizing agent, ^●^OH radicals, probably due to the Fenton reaction since the activation of molecular oxygen leads to the formation of H_2_O_2_. This could be the reason for the decrease in HMF selectivity in the aqueous medium, i.e., degradation of HMF and its product, such as DFF by ^●^OH. In the presence of a higher concentration of HMF (50 mM), the DFF production rate increased due to the increase in the number of R-OH functional-group-containing-molecules (HMF) in the reaction medium, which act as hydroxyl radical scavengers [42,43]. To evaluate the scavenger effect, a test using 10% ethanol as an additive in the aqueous medium was performed. There was a drop in conversion from 68.2 to 22.3% and an increase in selectivity from 18.8 to 31.8%; this result indicates that the presence of ethanol prevents the destruction of the substrate as well as its derivatives by EtOH. When a PHI(Fe) H_2_O/EtOH suspension was illuminated in the presence of alpha-phenyl N-tertiary-butyl nitrone (PBN) as a spin-trapping agent, the α-hydroxy ethyl (CH_3_^●^CHOH, a_N_ = 15.2 G and 3.6 G) spin adduct was readily detected by EPR (Figure S9a) [1], further corroborating this scavenger effect.

No EPR signal of any DMPO spin adduct was detected over a 0.1 wt% PHI(Fe) aqueous suspension (5 mM HMF) in the absence of O_2_; only an intense and sharp signal (Figure 5c) was observed, which has already been reported and is related to the accumulation of electrons in the polymeric matrix. Irradiation of the catalysts led to an increase in the EPR signal intensity with time without the formation of any DMPO spin adducts (Figure S9b). When AcN is used instead of water under Ar, a six-line signal (Figure 5d) was observed due to the formation of a DMPO-R spin adduct (a_N_ = 14.6 G and 21.0 G), overlapped with the paramagnetic signal of the carbon nitride [44,45]. The non-detection of DMPO-spin adducts in water suggests that HMF oxidation follows a different pathway in each solvent; it is possible that the intermediate in the aqueous medium could be an alkoxide radical rather than a carbon-centered one, as observed in acetonitrile, given the high instability of oxygen-centered spin adducts [46,47].

**Scheme 1:**
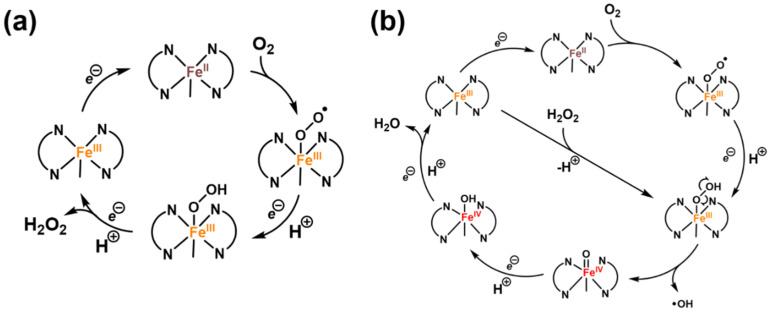
Proposed mechanism for (**a**) H_2_O_2_ production from O_2_ activation and (**b**) Fe^IV^=O and ^●^OH species production from O_2_ activation and H_2_O_2_ decomposition.

As mentioned before, H_2_O_2_ could be produced during photocatalytic activation of molecular oxygen in the presence of H^+^ donor molecules such as HMF and ethanol. Therefore, the detection and determination of H_2_O_2_ concentration during the photocatalytic reaction were performed for 0.1 wt% PHI(Fe) and PHI(Na) in 10% ethanol aqueous solution. A measure of 4.18 mM of H_2_O_2_ was detected over 0.1 wt% PHI(Fe), while for PHI(Na) it was only 1.01 mM. The formation of a higher H_2_O_2_ concentration over 0.1 wt% PHI(Fe) in an early stage of the reaction was reported by Teixera et al., which then decomposed to generate Fe^IV^=O groups [16]. In fact, the production of these species is quite common during H_2_O_2_ decomposition or O_2_ activation by N-coordinated Fe sites, and in both processes, hydroxyl radicals can be produced [48,49]. Based on the EPR results and the above-mentioned information, photocatalytic mechanisms were proposed to illustrate H_2_O_2_ production/decomposition, O_2_ activation and ^●^OH generation by Fe^3+^ sites in PHI (Scheme 1a,b). In pathway a (Scheme 1a), we assumed that highly dispersed Fe^3+^ sites are coordinated to the nitrogen ligand of PHI (simplified as a heme structure). Trapping the photoexcited electron by Fe^3+^ reduces it to Fe^2+^, which in turn favors, as in biological systems, molecular oxygen coordination and activation (chemisorption). The coordinated O_2_ is then reduced to ^●^OOH after abstracting proton-form HMF and forming Fe^3+^ subsequent addition of *e^-^*/H^+^ releases H_2_O_2_ to the reaction medium and restoring the photocatalytic cycle. In pathway b (Scheme 1b), the homolytic cleavage of Fe-OOH leads to the formation of ^●^OH and the Fe^IV^=O species via both O_2_ activation and H_2_O_2_ decomposition. The Fe^IV^=O complex is reduced to Fe^IV^-OH, which is subsequently reduced again and regenerates the Fe^3+^ active site species by releasing H_2_O. It is worth noting that electrons are supplied by the semiconductor and H^+^ is obtained from HMF. Generally, the oxidation of the HMF by the photogenerated holes should be more selective to produce DFF. However, due to the different pathways of nonselective ^●^OH radical generation as well as via Fenton reaction [50,51], the selectivity of highly active Fe-PHI is low. Therefore, conducting the photocatalytic reaction in ethanol, the selectivity increased due to its ^●^OH radical scavenging activity.

## 3. Materials and Methods

### 3.1. Poly(heptazine imide)-Based Material Syntheses

#### 3.1.1. PHI(Na) Synthesis

The PHI(Na) material was synthesized according to a previous method reported by Teixeira et al. [16]. A mixture of melamine (10 g) and sodium chloride (NaCl—100 g) was ground in a ball mill for 5 min with a frequency of 25 rpm·s^−1^. The solid mixture was placed in a porcelain crucible and heated up in an oven to 600 °C with a heating rate of 2.3 °C min^−1^ under a constant nitrogen flow (5 L min^−1^), then held at 600 °C for 4 h. After cooling down, the product was washed with deionized water (1 L) at 90 °C for 2 h to remove solubilized NaCl. Then, the yellow solid was isolated using centrifugation (9000 rpm, 5 min), washed abundantly with deionized water and dried overnight in a vacuum oven (10 mbar) at 60 °C.

#### 3.1.2. PHI(TM) Photocatalysts

The PHI(TM) materials were prepared using a previously reported cation exchange method in which 200 mg of as-synthesized PHI(Na) was mixed with 4 mL of 20, 10, 1 mM metal chloride (MCl_X_) solution vigorously to homogenize the suspension and obtain 2, 1 and 0.1 metals wt% PHI, respectively. After that, the suspensions were sonicated for 1 h, centrifuged (9000 rpm, 5 min) and washed with deionized water (12 mL). Finally, the PHI(TM) were dried overnight in a vacuum oven (10 mbar) at 60 °C.

### 3.2. Ex and In Situ Characterizations

The metal analysis of Na, Fe, Co, Ni and Cu was determined by inductively coupled plasma optical emission spectroscopy (ICP-OES) using an Optima 8000 ICP-OES from PerkinElmer. The infrared spectrum of PHI(Na) was recorded from 500 to 4000 cm^−1^ (with a spectral resolution of 0.5 cm^−1^) by pressing the powder onto a diamond crystal placed on an ATR (attenuated total reflectance) module installed on a Nicolet 6700 FTIR spectrophotometer with an MCT detector. The thermogravimetric analysis (TGA) was performed on a Thermo Microbalance TG 209 F1 Libra (Netzsch, Selb, Germany); the thermogram was acquired in the temperature range of 25–900 °C using a flow of 20 mL min^−1^ of synthetic air at a heating rate of 10 °C min^−1^ in an alumina crucible. The transmission electron microscopy images as well as the diffraction by selected area (SAED) PHI(Na) were obtained using a TECNAI G2-20 Super Twin FEI 200 kV. Powder X-ray diffraction data (XRD) were obtained using a Rigaku SmartLab Diffractometer (Japan, Cu Kα of 0.154 nm). The N_2_ adsorption/desorption at isotherm at 77 K measurement for PHI(Na) was performed on a Quantachrome Quadrasorb SI porosimeter instrument. The sample was degassed at 150 °C for 20 h under vacuum. The Brunauer–Emmet–Teller (BET) method was used for analyzing the surface area and the BJH model was used to determine the pore size distribution. The UV–Vis Kubelka–Munk spectra were obtained using Shimadzu UV 2600 in diffuse reflectance mode.

Powder EPR measurements: The EPR measurements of PHI-based photocatalysts were performed by weighing each material in a conventional quartz EPR tube, which was then inserted into a front-gridded cavity for in situ irradiation; the sample and the spectra were recorded using a Bruker EMX CW-micro X-band EPR spectrometer equipped with the high-sensitivity resonator ER 4119 HS. A 300 W Xe lamp with a 420 nm installed cut-off filter was used for time-resolved visible-light in situ irradiation measurements; these spectra were obtained by recording the first spectrum in the dark, then illuminating for 10 min, collecting a spectrum every 30 s, before finally turning off the lamp and collecting again every 30 s for 10 min (in the dark). The EPR figures showing the spectra over time were obtained by subtracting the spectra obtained under light and after switching off the lamp from the initial spectrum of the photocatalyst (which was recorded in the dark). The trapped electron plots were obtained by taking the amplitudes of the main signals of the spectra in the dark and dividing them by the last spectrum under irradiation. All EPR data were normalized by the mass of the previously weighed solid.

Spin trapping method: Spin-trapping tests were performed by weighing 2 mg of photocatalyst in a vial and suspending this solid in 400 µL of 5 mM HMF solution (same ratio used in photocatalytic tests) followed by purging the mixture Ar or O_2_. In this suspension, 88.5 µmol of the DMPO spin-trapping agent (5,5-dimethyl-1-pyrroline-N-oxide) was added and 50 µL was extracted from this system through a capillary, which was placed in an EPR tube and inserted into the cavity of the spectrometer. The spectra were acquired during irradiation of the capillary on the same apparatus described in the previous section. The spin adducts were simulated using an EasySpin MATLAB toolbox [52].

Time resolved Photoluminescence: Time-resolved photoluminescence (TRPL) measurements were performed using a fluorescence lifetime spectrometer (FluoTime 250, PicoQuant) equipped with a PDL 800-D picosecond pulsed diode laser drive. The analyses were performed in a solid state with an excitation wavelength of 375 nm. The excitation was recorded using a pulsed laser diode at 30 nJ cm^−2^ (LDH-P-C405, PicoQuant GmbH). The emission was recorded at 460 nm. Raw decay data presented as the logarithm of photon counts versus time were analyzed with data analysis software from PicoQuant GmbH (Berlin, Germany). The acquired PL lifetime decay curve was fitted tri-exponentially, as reported previously, for carbon-nitride-based materials using the following equation:

I(t) = *A*_1_e^−t/τ1^ + *A*_2_e^−t/τ2^ + *A*_3_e^−t/τ3^ (1)

where *A*_1_, *A*_2_ and *A*_3_ represent the normalized amplitudes of each decay component and τ_1_, τ_2_ and τ_3_ are values of the lifetime components, respectively.

### 3.3. Photocatalytic Tests and Reaction Medium Analysis

Photocatalytic reactions were performed by adding 10 mg of material into a 10 mL glass tube containing 2 mL of 5 mM or 50 mM HMF solution (water or organic). In the case of conducting the catalytic reactions at different pH levels, HCl and NaOH solutions were used to adjust the pH to the acid and basic mediums, respectively. The tube was then left for 5 min in the ultrasound and then the suspension was saturated with O_2_ by bubbling it for 1 min, followed by covering the top part of the reactor with an O_2_ balloon to keep the suspension saturated with the gas. Finally, the reactors were illuminated by a blue LED strip and stirred using a magnetic bar at 600 rpm. A 1 mL aliquot was extracted at the end of the reaction, filtered through a 0.45 µm membrane and inserted into a vial that was injected into an Agilent HPLC model 1260 Infinity II equipped with a DAD detector. The conditions used to detect the products were 5 mM H_2_SO_4_ mobile phase, a 0.6 mL·min^−1^ pumping flow, 2.4 µL volume injection and 50 min chromatographic run, and the products were monitored using 280 nm fixed wavelength. Figure S1 shows the chromatographic separation of HMF and its oxidation products. Calibration curves were prepared for the quantification of substrate and products as well as to obtain the conversion and selectivity results.

### 3.4. Hydrogen Peroxide Production

The hydrogen peroxide concentration was determined by a colorimetric method using ammonium titanyl oxalate monohydrate [16,32]. For this, 2 mL of an aqueous solution with 10% (*v*/*v*) of ethanol as a hole scavenger was used to prepare a suspension with the desired photocatalysts, then it was irradiated for 2 h and 1 mL of the sample was added to 3 mL of an acidified solution of ammonium titanyl oxalate monohydrate (8.33 g/L). The absorbance of the peroxo complex, formed by the reaction between Ti(IV) and hydrogen peroxide, was measured at 400 nm using an Avantes UV–Vis spectrophotometer model AvaSpec-2048 (equipped with an Ava Light-DH-S-BAL light source and 0.5 cm optical pathway glass fiber probe), and quantification was performed using a linear calibration curve ranging from 0.3 to 4 mM.

## 4. Conclusions

In conclusion, different Fe-, Co-, Ni- and Cu-supported catalysts on sodium Poly(heptazine imide), PHI(Na), were prepared using the cation exchange method and tested using the photocatalytic aerobic oxidation of HMF to DFF in an aqueous system under blue LED irradiation (λ_max_ = 456 nm). The best yield of DFF was obtained over 0.1 wt% PHI(Fe); therefore, the effect of metal loading, solvents, pH, atmosphere, and additives has been explored for this catalyst. Furthermore, water proved to be the best solvent for obtaining a high rate of DFF (393 μmol·h^−1^·g^−1^); however, the generation of highly active and nonselective ^●^OH radical, oxidized HMF and its products over time was observed. The generation of ^●^OH radical was attributed to the production and decomposition of H_2_O_2_ during the photoredox process. A strategy adopted to reduce DFF degradation in the medium was to increase the HMF concentration (from 5 to 50 mM). Therefore, it was possible to obtain a DFF production rate of 393 μmol·g^−1^·h^−1^, the highest value observed in literature for an aqueous system under visible light irradiation. Control tests showed that there is no conversion in the absence of electron acceptors, indicating that the presence of O_2_ is essential to avoid the recombination of photogenerated charges.

## Data Availability

The data presented in this study are available on request from the corresponding author. The data are not publicly available due to privacy.

## Supplementary Materials

The following supporting information can be downloaded at https://www.mdpi.com/article/10.3390/molecules28248077/s1: Figure S1. Chromatographic separation of HMF and its oxidation products using the method described in the experimental section. Figure S2. (a), (b) HR-TEM images of PHI(Na), (c) SAED of (b). Figure S3. XRD powder pattern for PHI(Na), PHI(Fe) 1%, PHI(Fe) 2%, PHI(Ni) 1% and PHI(Ni) 2%. Figure S4. Difference between (Na)PHI suspension in water (H2O) and acetonitrile (AcN). Figure S5. Chromatographic run of the sample collected after 2h of reaction overlapped with formic acid standard. Figure S6. (a) Survey, high resolution spectra of (b) C 1s and (c) N 1s bind energy range for PHI(Na). Figure S7. (a) Survey, high resolution spectra of (b) C 1s, (c) N 1s and (d) Fe 2p binding energy range for 0.1 wt% PHI(Fe). Figure S8. EPR spectra over time recorded in light ON for 10 min and then light OFF for 10 min obtained from (a) 1 wt% Fe PHI (b) 2 wt% Fe PHI, (c) TRPL for PHI(Na) and 0.1 wt% Fe PHI aqueous 5 mM HMF suspensions saturated with O2 and (d) EPR spectra recorded for PHI(Na) and PHI(Fe) in dark showing the intrinsic signal of carbon nitride at g = 2.004. Figure S9. (a) Experimental and simulated spectra of α-hydroxy ethyl spin adduct of PBN obtained in 5mM HMF with 10% EtOH aqueous solution with 0.1 wt% Fe PHI, irradiated by a Xenon lamp with a >420 nm cut off filter; (b) 5mM HMF suspension of 0.1 wt% PHI(Fe) in Ar atmosphere irradiated over time. Table S1. Theoretical amount of metal compared with experimental result of ICP-OES. Table S2. DFF production rate comparing this work with literature results on HMF oxidation in aqueous medium in O2 atmosphere. References [4,24,53,54,55,56,57] are cited in the supplementary materials.
