# Supplementary material for: Aerobic Oxidation of 5-Hydroxymethylfurfural (HMF) in Aqueous Medium over Fe-Doped-Poly(heptazine imide) Photocatalysts: Unveiling the Bad Role of Hydroxyl Radical Generation on the Catalytic Performance
Source: Molecules. 2023 Dec 14;28(24):8077. doi: 10.3390/molecules28248077 (PMC10745455; doi:10.3390/molecules28248077)
Supplement: Supplementary file 1 [file molecules-28-08077-s001.zip › molecules-2734022-supplementary.pdf]

Aerobic Oxidation of 5-Hydroxymethylfurfural (HMF) in Aqueous Medium over Fe-Doped-Poly(heptazine imide) Photocatalysts: Unveiling the Bad Role of Hydroxyl Radical Generation on the Catalytic Performance

José B. G. Filho[a,b], Ingrid F. Silva[c], Mamduoh Alafandi[a], Jabor Rabeah[a]*

[a]Leibniz Institute for Catalysis (LIKAT Rostock), D-18059, Rostock, Germany

[b]Chemistry Department, Federal University of Minas Gerais (UFMG), 31270-901 Belo Horizonte, MG, Brazil

[c]Department of Colloid Chemistry, Max Planck Institute of Colloids and Interfaces, Potsdam Science Park, Am Mühlenberg 1, D-14476 Potsdam, Germany

*Corresponding author: Jabor.Rabeah@catalysis.de

Table of Contents

Figures and related texts....................2-7

Tables....................8

References....................9

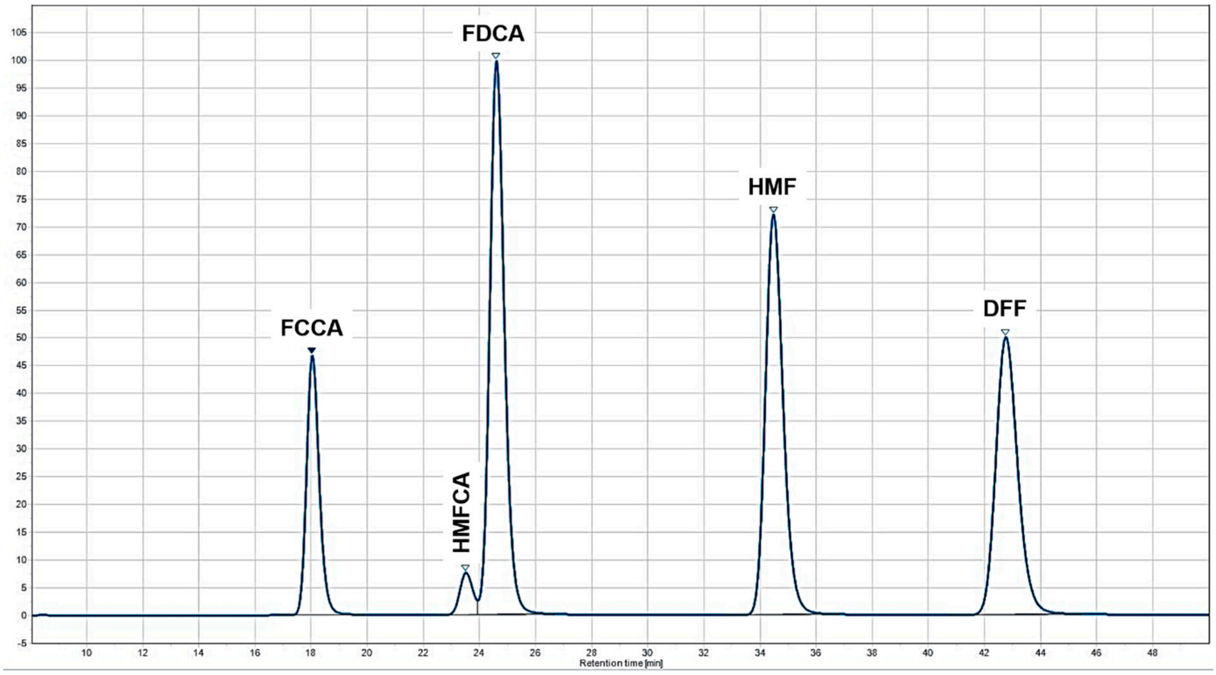

Figure S1. Chromatographic separation of HMF and its oxidation products using the method described in the experimental section.

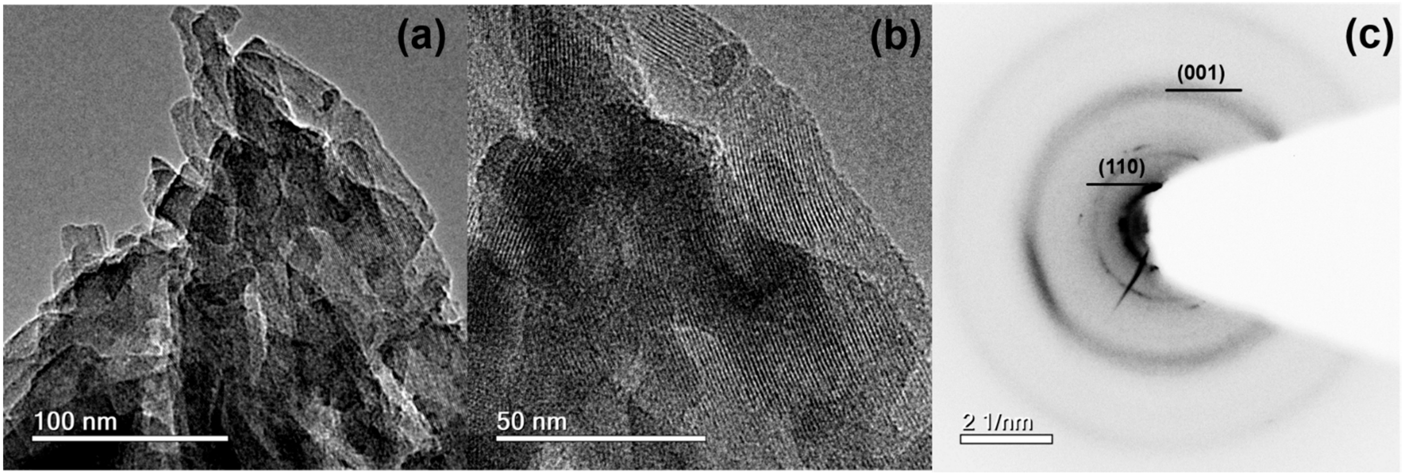

Figure S2. (a), (b) HR-TEM images of PHI(Na), (c) SAED of (b).

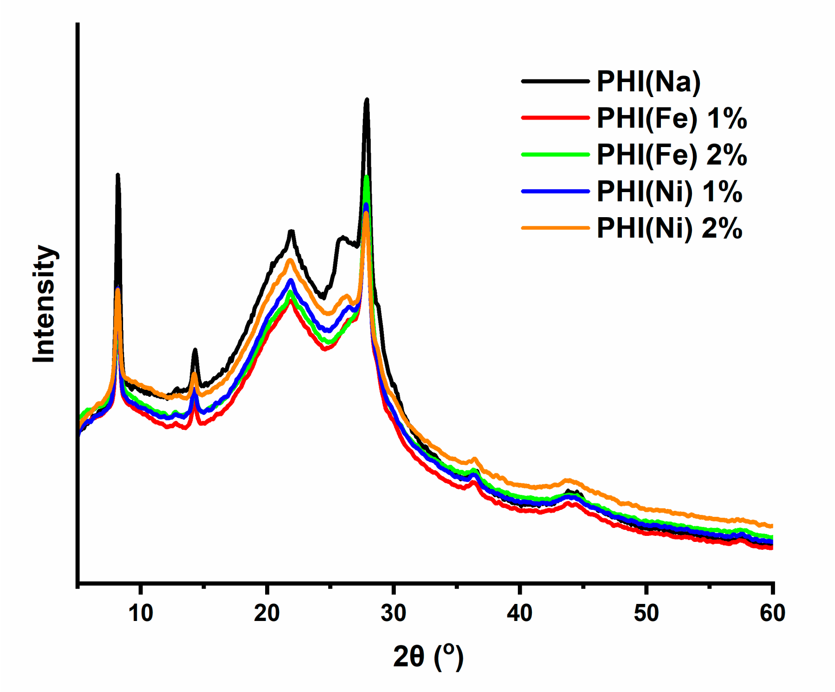

Figure S3. XRD powder pattern for PHI(Na), PHI(Fe) 1%, PHI(Fe) 2%, PHI(Ni) 1% and PHI(Ni) 2%.

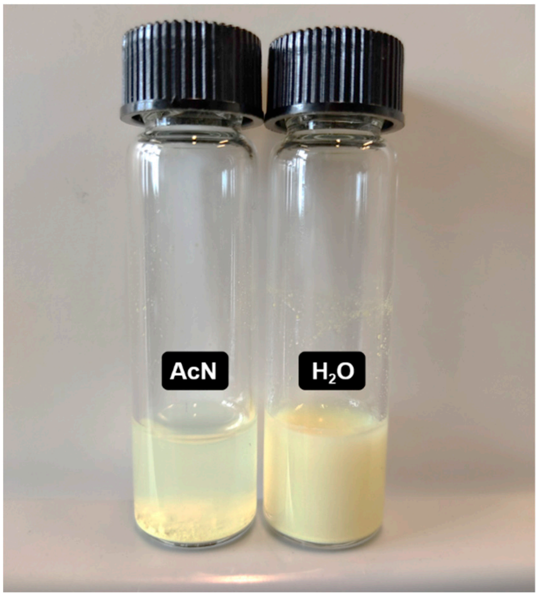

Figure S4. Difference between (Na)PHI suspension in water (H_2O) and acetonitrile (AcN).

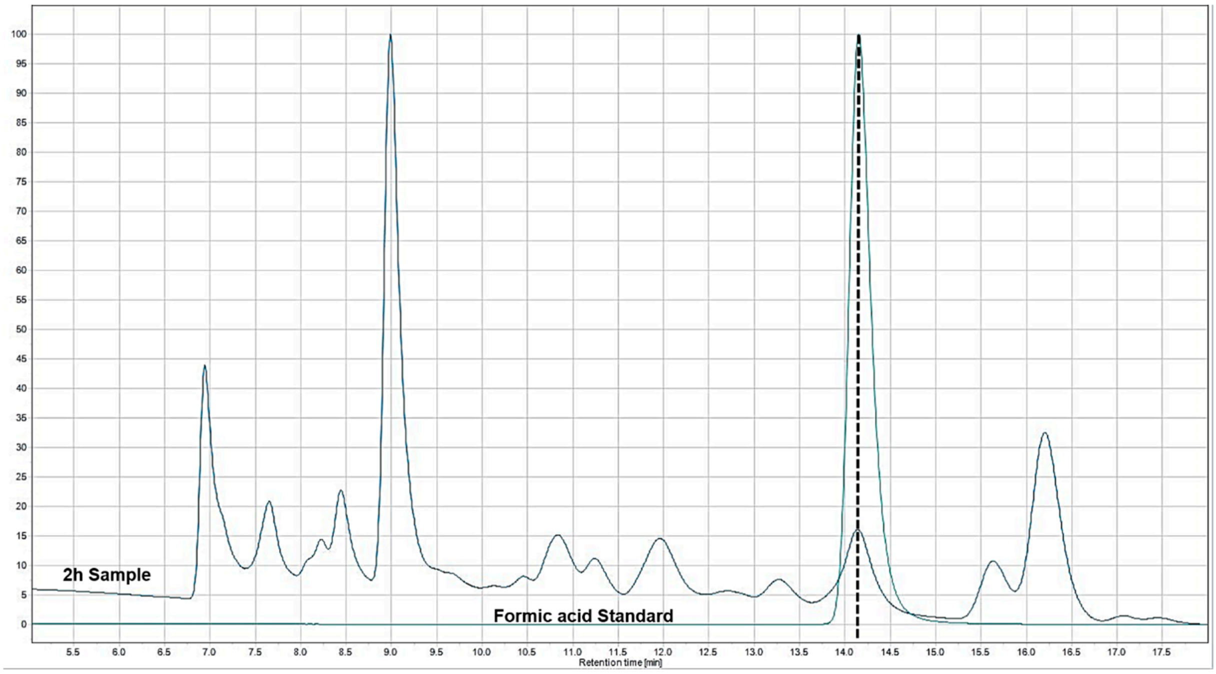

Figure S5. Chromatographic run of the sample collected after 2h of reaction overlapped with formic acid standard.

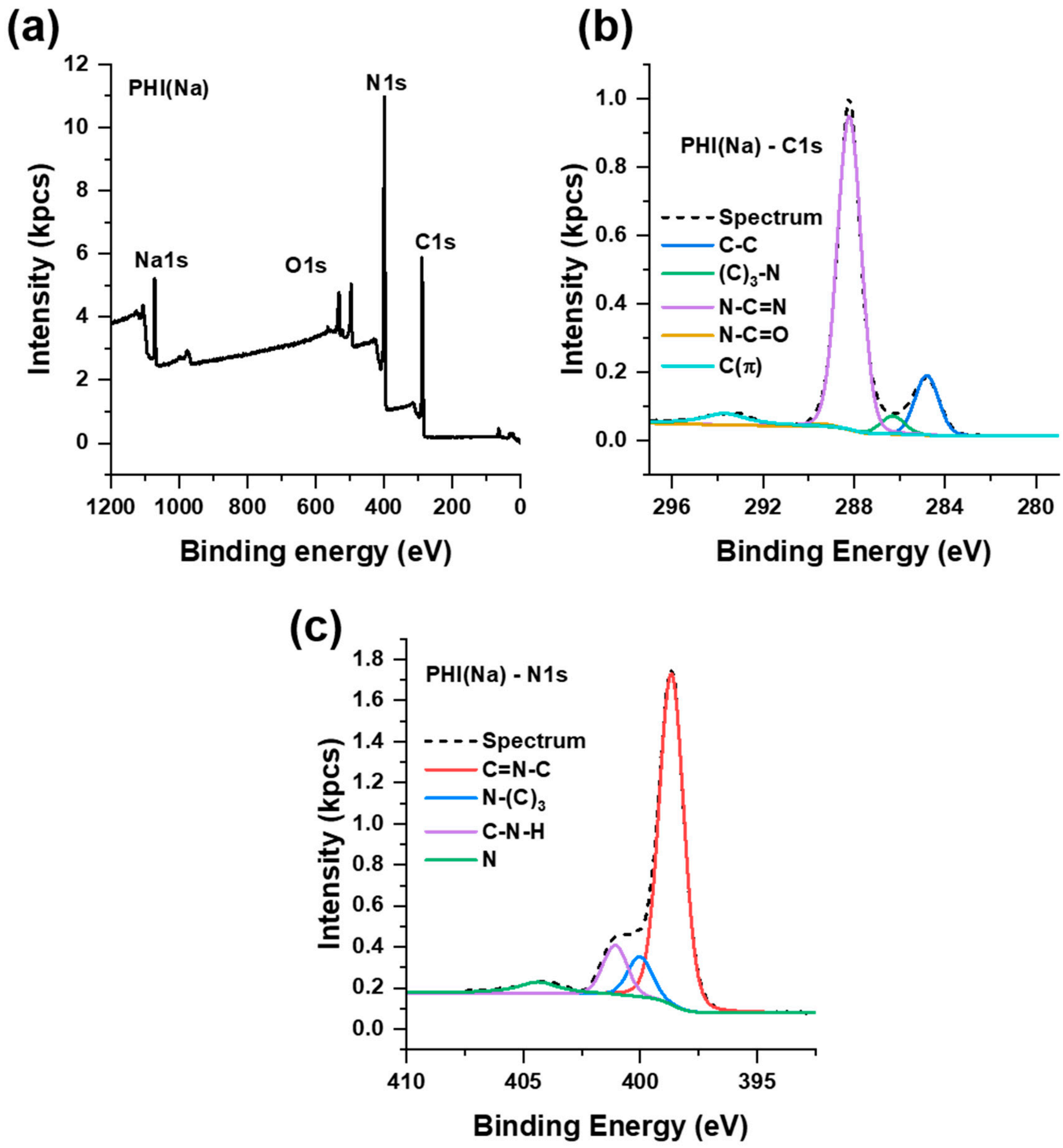

Figure S6. (a) Survey, high resolution spectra of (b) C 1s and (c) N 1s bind energy range for PHI(Na)

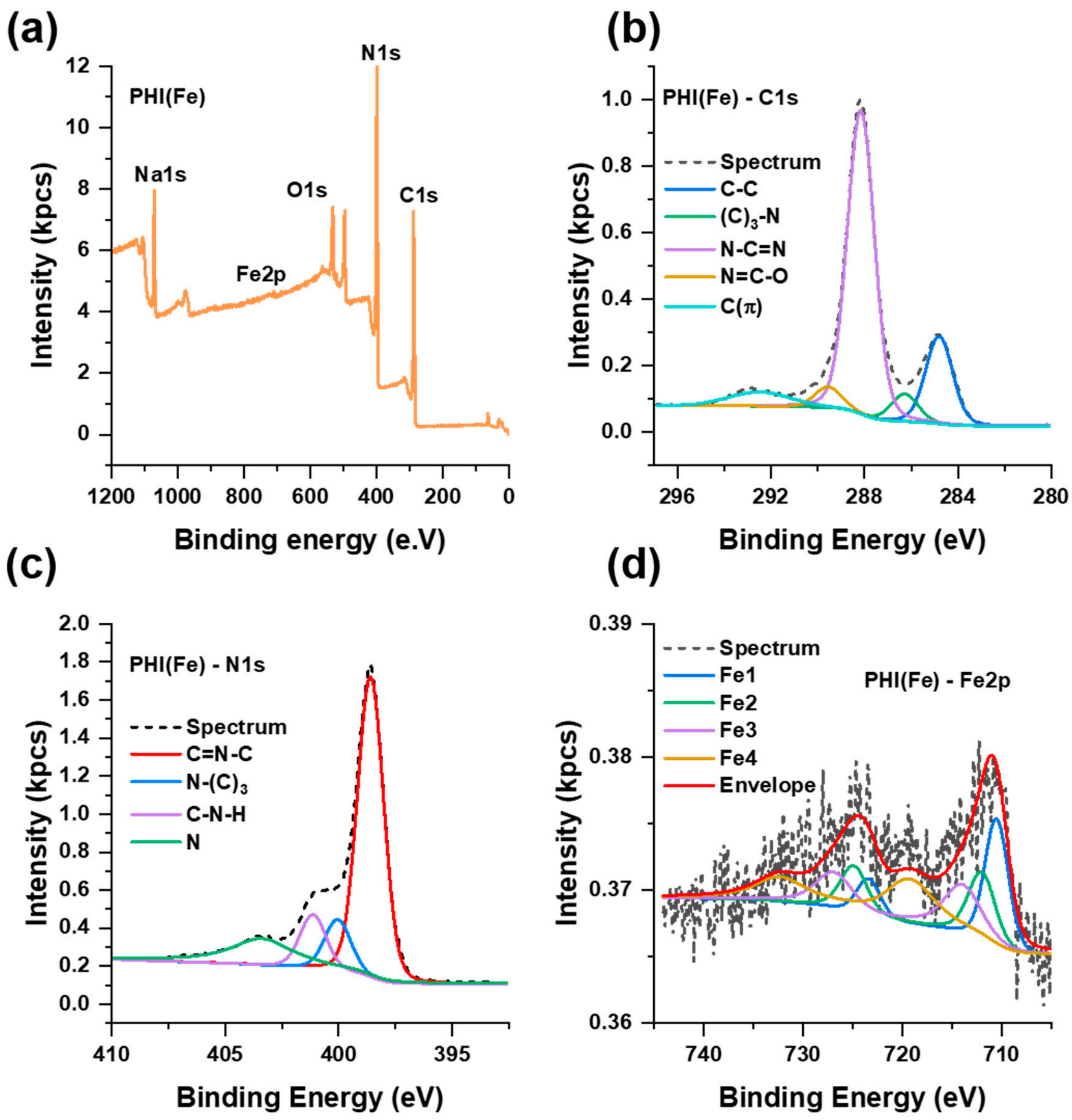

Figure S7. (a) Survey, high resolution spectra of (b) C 1s, (c) N 1s and (d) Fe 2p binding energy range for 0.1 wt% PHI(Fe).

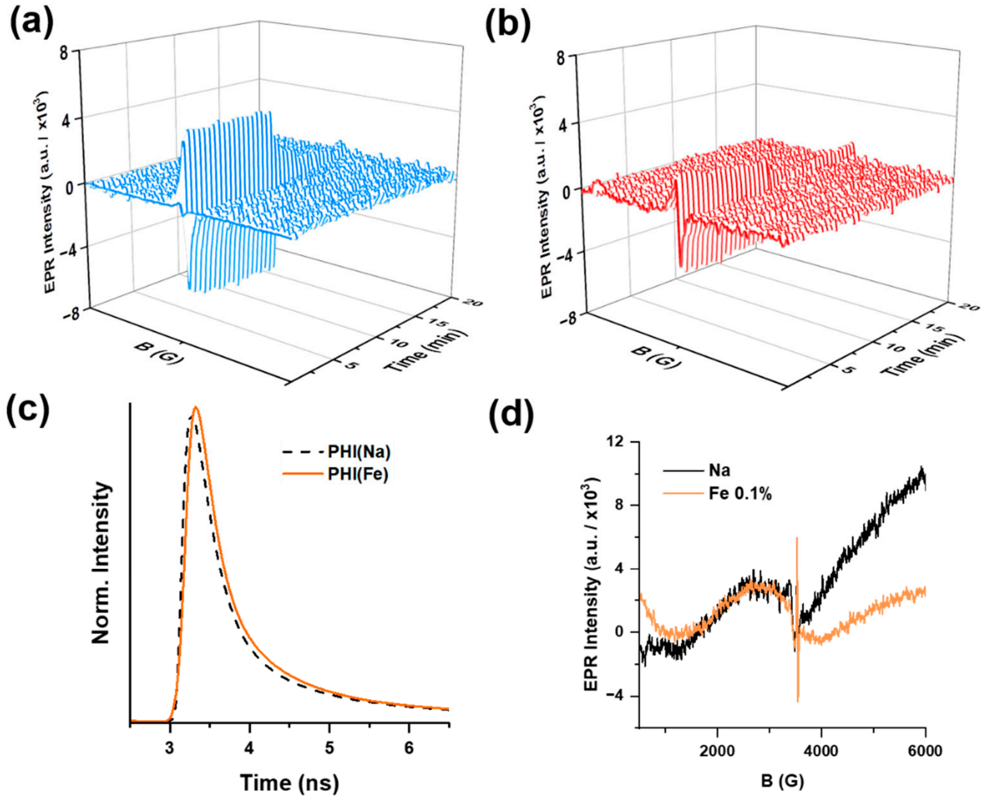

Figure S8. EPR spectra over time recorded in light ON for 10 min and then light OFF for 10 min obtained from (a) 1 wt% Fe PHI (b) 2 wt% Fe PHI, (c) TRPL for PHI(Na) and 0.1 wt% Fe PHI aqueous 5 mM HMF suspensions saturated with O_2 and (d) EPR spectra recorded for PHI(Na) and PHI(Fe) in dark showing the intrinsic signal of carbon nitride at g = 2.004.

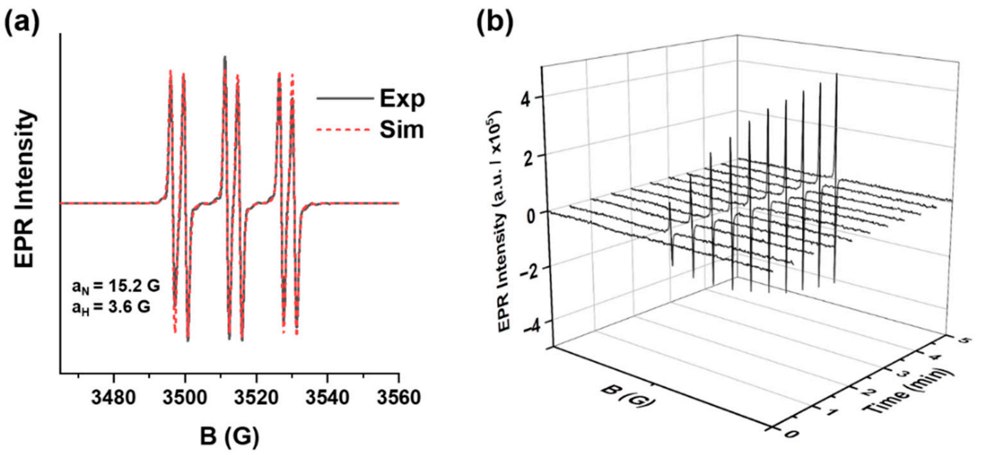

Figure S9. (a) Experimental and simulated spectra of α-hydroxy ethyl spin adduct of PBN obtained in 5mM HMF with 10% EtOH aqueous solution with 0.1 wt% Fe PHI, irradiated by a Xenon lamp with a >420 nm cut off filter; (b) 5mM HMF suspension of 0.1 wt% PHI(Fe) in Ar atmosphere irradiated over time.

Table S1. Theoretical amount of metal compared with experimental result of ICP-OES.

Photocatalyst	Metal (% m/m)	
	Theoretical	Experimental
PHI(Fe)	0.1	0.1
PHI(Co)	0.1	0.06
PHI(Cu)	0.1	0.3
PHI(Fe)	0.1	0.1
PHI(Fe) 1%	1.0	0.9
PHI(Fe) 2%	2.0	2.3
PHI(Ni) 1%	1.0	1.0
PHI(Ni) 2%	2.0	2.1

Table S2. DFF production rate comparing this work with literature results on HMF oxidation in aqueous medium in O_2 atmosphere.

Photocatalyst	DFF (µmol.g^{-1}.h^{-1})	Ref.
PHI(Fe)	393.0	This work
Ti_3C_2Fx MXene/$CdIn_2S_4$	125.6	[1]
MoS2/CdIn2S4 (12.5%)	49.2	[2]
CTF Th@SBA-15	190.0	[3]
g-C_3N_4	26.7	[4]
1.5% Fe (III)/BMO	77.7	[5]
12% Bi2WO6/mpg–C3N4	0.2	[6]
Zn0.5Cd0.5S/1%MnO2	303.9	[7]

References

[1] M. Zhang, Y. Zhang, L. Ye, Z. Yu, R. Liu, Y. Qiao, L. Sun, J. Cui, X. Lu, In situ fabrication Ti3C2Fx MXene/CdIn2S4 Schottky junction for photocatalytic oxidation of HMF to DFF under visible light, Appl. Catal. B Environ. 330 (2023) 122635. https://doi.org/10.1016/j.apcatb.2023.122635.

[2] Q. Zhu, Y. Zhuang, H. Zhao, P. Zhan, C. Ren, C. Su, W. Ren, J. Zhang, D. Cai, P. Qin, 2,5-Diformylfuran production by photocatalytic selective oxidation of 5-hydroxymethylfurfural in water using MoS2/CdIn2S4 flower-like heterojunctions, Chinese J. Chem. Eng. 54 (2023) 180–191. https://doi.org/10.1016/j.cjche.2022.04.018.

[3] C. Ayed, W. Huang, G. Kizilsavas, K. Landfester, K.A.I. Zhang, Photocatalytic Partial Oxidation of 5-Hydroxymethylfurfural (HMF) to 2,5-Diformylfuran (DFF) Over a Covalent Triazine Framework in Water, ChemPhotoChem. 4 (2020) 571–576. https://doi.org/10.1002/cptc.202000070.

[4] Q. Wu, Y. He, H. Zhang, Z. Feng, Y. Wu, T. Wu, Photocatalytic selective oxidation of biomass-derived 5-hydroxymethylfurfural to 2,5-diformylfuran on metal-free g-C3N4 under visible light irradiation, Mol. Catal. 436 (2017) 10–18. https://doi.org/10.1016/j.mcat.2017.04.012.

[5] J. Xue, C. Huang, Y. Zong, J. Gu, M. Wang, S. Ma, Fe (III)-grafted Bi2MoO6 nanoplates for enhanced photocatalytic activities on tetracycline degradation and HMF oxidation, Appl. Organomet. Chem. 33 (2019) 1–10. https://doi.org/10.1002/aoc.5187.

[6] L. Cheng, D. Huang, Y. Zhang, Y. Wu, Photocatalytic selective oxidation of HMF to DFF over Bi2WO6/mpg–C3N4 composite under visible light, Appl. Organomet. Chem. 35 (2021) 1–9. https://doi.org/10.1002/aoc.6404.

[7] S. Dhingra, T. Chhabra, V. Krishnan, C.M. Nagaraja, Visible-Light-Driven Selective Oxidation of Biomass-Derived HMF to DFF Coupled with H2Generation by Noble Metal-Free Zn0.5Cd0.5S/MnO2Heterostructures, ACS Appl. Energy Mater. 3 (2020) 7138–7148. https://doi.org/10.1021/acsaem.0c01189.
